# Minimal invasive aortic valve replacement: associations of radiological assessments with procedure complexity

**DOI:** 10.1186/s13019-019-0997-5

**Published:** 2019-10-12

**Authors:** Bruce R. Boti, Vikash G. Hindori, Emilio L. Schade, Athina M. Kougioumtzoglou, Eva C. Verbeek, Annet Driessen-Waaijer, Riccardo Cocchieri, Bas A. J. M. de Mol, Nils R. Planken, Abdullah Kaya, Henk A. Marquering

**Affiliations:** 1 Department of Biomedical Engineering & Physics, Amsterdam UMC, location AMC, Meibergdreef 9, 1105 AZ Amsterdam, The Netherlands; 2 Department of Radiology and Nuclear Medicine, Amsterdam UMC, location AMC, Meibergdreef 9, 1105 AZ Amsterdam, The Netherlands; 3 Department of Cardiothoracic Surgery, Amsterdam UMC, location AMC, Meibergdreef 9, 1105 AZ Amsterdam, The Netherlands; 4 Department of Cardiothoracic Surgery, OLVG, location East, Oosterpark 9, Amsterdam, 1091 AC The Netherlands; 5 Department of Radiology, OLVG, location East, Oosterpark 9, Amsterdam, 1091 AC The Netherlands

**Keywords:** Aortic valve replacement, Minimal invasive surgery, Imaging, Computed tomography

## Abstract

**Objectives:**

Limited aortic annulus exposure during minimal invasive aortic valve replacement (mini-AVR) proves to be challenging and contributes to procedure complexity, resulting in longer procedure times. New innovations like sutureless valves have been introduced to reduce procedure complexity. Additionally, preoperative imaging could also contribute to reducing procedure times. Therefore, we hypothesize that Computed Tomography (CT)-image based measurements are associated with mini-AVR complexity.

**Methods:**

One hundred patients who underwent a mini-sternotomy and had a preoperative CT scan were included. With a CT-based mini-AVR planning tool, we measured access distance, access angle, annulus dimensions, and calcium volume. The associations of these measurements with cardiopulmonary bypass (CPB) time and aortic cross-clamp (AoX) time were assessed using univariable and multivariable regression models. In the multivariable models, these measurements were adjusted for age and suture technique.

**Results:**

In the univariable regression models, calcium volume and annulus dimensions were associated with longer CPB and AoX time. After adjusting for age and suture technique, increasing calcium volume was still associated with longer CPB (adjusted β-coefficient 0.002, 95%-CI (0.005, 0.019), *p*-value = 0.002) and AoX time (adjusted β-coefficient 0.010, 95%-CI (0.004, 0.016), *p*-value = 0.002). However, after adjusting for these confounders, the association between annulus dimensions and procedure times lost statistical significance.

**Conclusion:**

Increase in calcium volume are associated with longer CPB and AoX times, with age and sutureless valve implantation as independent confounders. In contrast to previous studies, access angle was not associated with procedure complexity.

## Introduction

Surgical valve replacement is standard treatment of severe aortic stenosis (AS). Minimally invasive aortic valve replacement (mini-AVR) is an alternative to conventional open-heart surgery to treat severe AS. Mini-AVR aims to minimize the degree of surgical intrusiveness by operating through a smaller incision (also known as ‘minimal access AVR’). Nevertheless, this procedure can still be considered an invasive surgical procedure. Because the term “Mini-AVR” has achieved common acceptance, we opted to use this as well in the remainder of this manuscript. Compared to conventional aortic valve replacement (CAVR), mini-AVR has shown to reduce transfusion incidences, postoperative pain, ICU stay, hospitalization, renal failure, and wound infection [1–3]. On the other hand, the limited exposure to the heart during mini-AVR makes myocardial protection and de-airing more challenging. Recent randomized controlled trials have shown that mini-sternotomy did not result in shorter hospital stay, faster recovery, improved survival, or less transfusion of blood compared to CAVR [4, 5]. In addition, mini-AVR has also been associated with increased cardiopulmonary bypass (CPB) time, aortic cross-clamp (AoX) time, overall operating time, and higher costs [6]. Several studies have demonstrated that prolonged CPB time and AoX time is associated with post-operative morbidity and mortality in both low- and high-risk cardiac patients [7–10]. An important advancement of mini-AVR procedures was the introduction of sutureless valves. Multiple studies have demonstrated that the use of sutureless valves reduces CPB and AoX time and are therefore associated with less post-operative complications [11–14].

To further improve mini-AVR procedures, preoperative imaging can be used to support surgical planning to decrease procedure complexity. 3D reconstructed Computer Tomography (CT)-images are commonly used for this goal, either for visual evaluation or for measuring distances between the incision and aortic valve [15–19].

In a recent study, it was found that the access angle, which is the angle between the aortic root and the incision at the annulus, was significantly associated with annulus access difficulty, CPB time, and AoX time in patients treated through a mini-sternotomy [20]. Other radiological characteristics that have been suggested to be associated with procedure complexity are the diameter of the aortic valve annulus, the distance from the ascending aorta to the sternum, and the extent of calcifications in the aortic valve and the ascending aorta [21, 22]. The reported association between access angle and procedural complexity was based on a relatively small patient population, which limited reliable statistical analysis for additional CT-based measures.

The primary purpose of this study is to determine whether CT-image based measurements are associated with mini-AVR surgical times. Additionally, we evaluate whether this hypothesized association of CT-image based parameters with the outcomes differ between sutured and sutureless valve replacement surgeries.

## Methods

### Study population

All patients that underwent mini-AVR at two institutes (Amsterdam UMC, location Academic Medical Center, The Netherlands; Onze Lieve Vrouwe Gasthuis, The Netherlands) and had a preoperative CT-scan between December 2014 and March 2018 were included in this study. The CT-imaging was not part of the treatment selection. Baseline characteristics, intraoperative data and postoperative outcomes were collected. Imaging kilovoltage ranged from 70 to 120 kV. The chest and abdomen were scanned using 1 bolus of contrast Iomeron 400 (Bracco Imaging SpA, Milan, Italy) ranging from 80 to 120 ml intravenously infused at a rate of 3.5–5.0 ml/s. In case patients had both CT and CT Angiography (CTA) acquisitions, the latter was used because of the higher contrast between the aortic root and surrounding structures. In case of dynamic CTA’s, the images acquired at 70% of the cardiac cycle (mid-diastole) were selected because at this phase the aortic valve is closed [20]. The image volumes contained approximately 400–900 slices, and each slice in a volume contained 512 × 512 isotropic pixels with a 16-bit depth. The slice thickness for the data sets ranged from 0.45–3.0 mm. The institutional review board approved the study design and waived informed consent since solely data obtained in the context of clinical care was utilized.

### Surgical technique

All patients in this study underwent a mini-sternotomy performed by one of three operators (A.K., V.H. and R.C.). This approach was executed through a J-shaped incision starting from the sternal angle (manubriosternal joint) moving caudally for 4 cm in the sternum body to the 3rd intercostal space. After establishing cardiopulmonary bypass, the left ventricle was vented, and the aorta was cross-clamped to subsequently apply cardioplegia to stop the heart. The diseased valve was sharply excised and surrounding calcium was removed, after which a prosthetic aortic valve was implanted. The implanted valves were either manually sutured or mechanically fastened using the Cor-Knot device (LSI Solutions, Victor, NY, USA). Additionally, sutureless Perceval aortic bioprostheses (Sorin, Saluggia, Italy) were implanted.

### CT-based mini-AVR planning tool

The mini-AVR planning tool is based on the 3mensio structural heart software (Pie Medical Imaging, Maastricht, The Netherlands) with additional options implemented for mini-AVR procedures. The tool allows the measurements of distances, angles, aortic valve dimensions, and calcifications. Furthermore, the tool automatically detects the aortic root, aortic annulus, and sinotubular junction and segments the ribcage in the CT scan. Additionally, the user can use a 3D probe to select an incision location on the 3D rendered chest cage for local measurements [20].

**Fig. 1:**
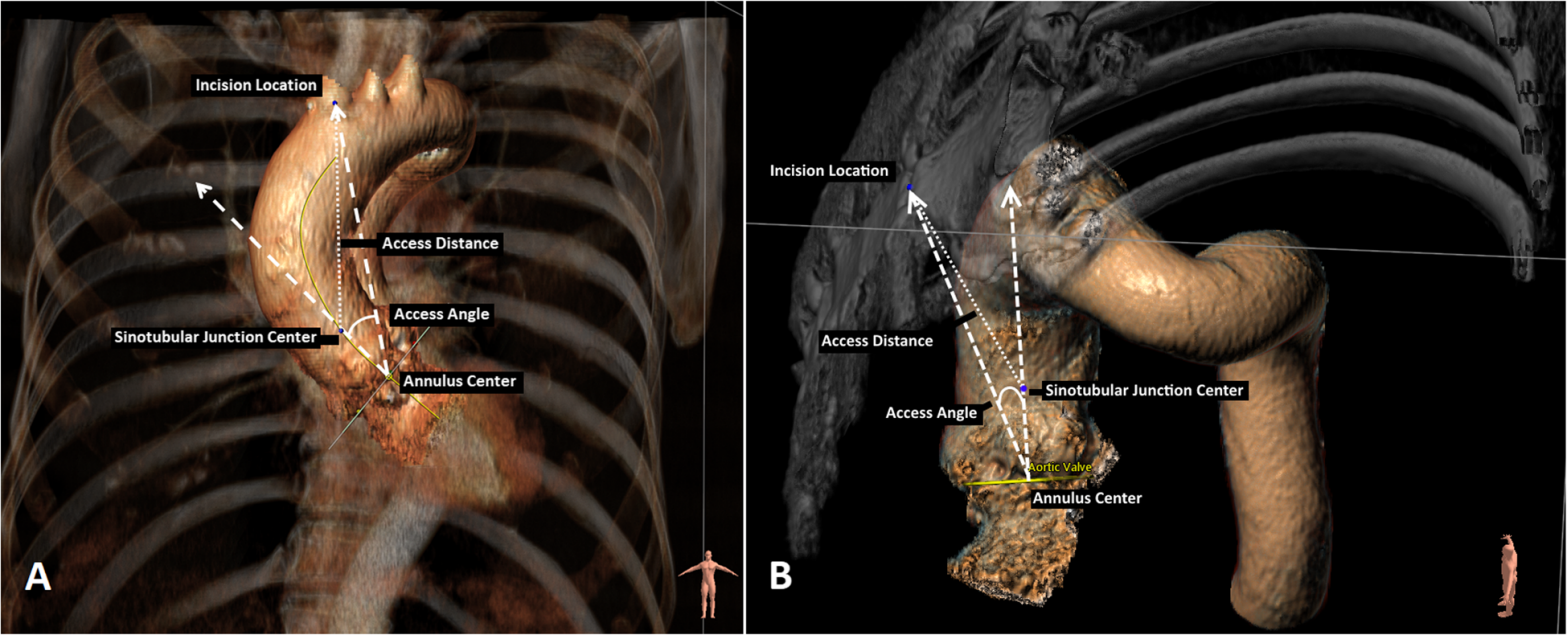
**a** Anterior (surgical) view of the aorta and ribcage. **b** Laterosuperior view of the aorta and partial rib cage. The access distance and access angle are determined based on the location of three landmarks: aortic annulus center, sinotubular junction and incision location (manubriosternal joint)

**Fig. 2:**
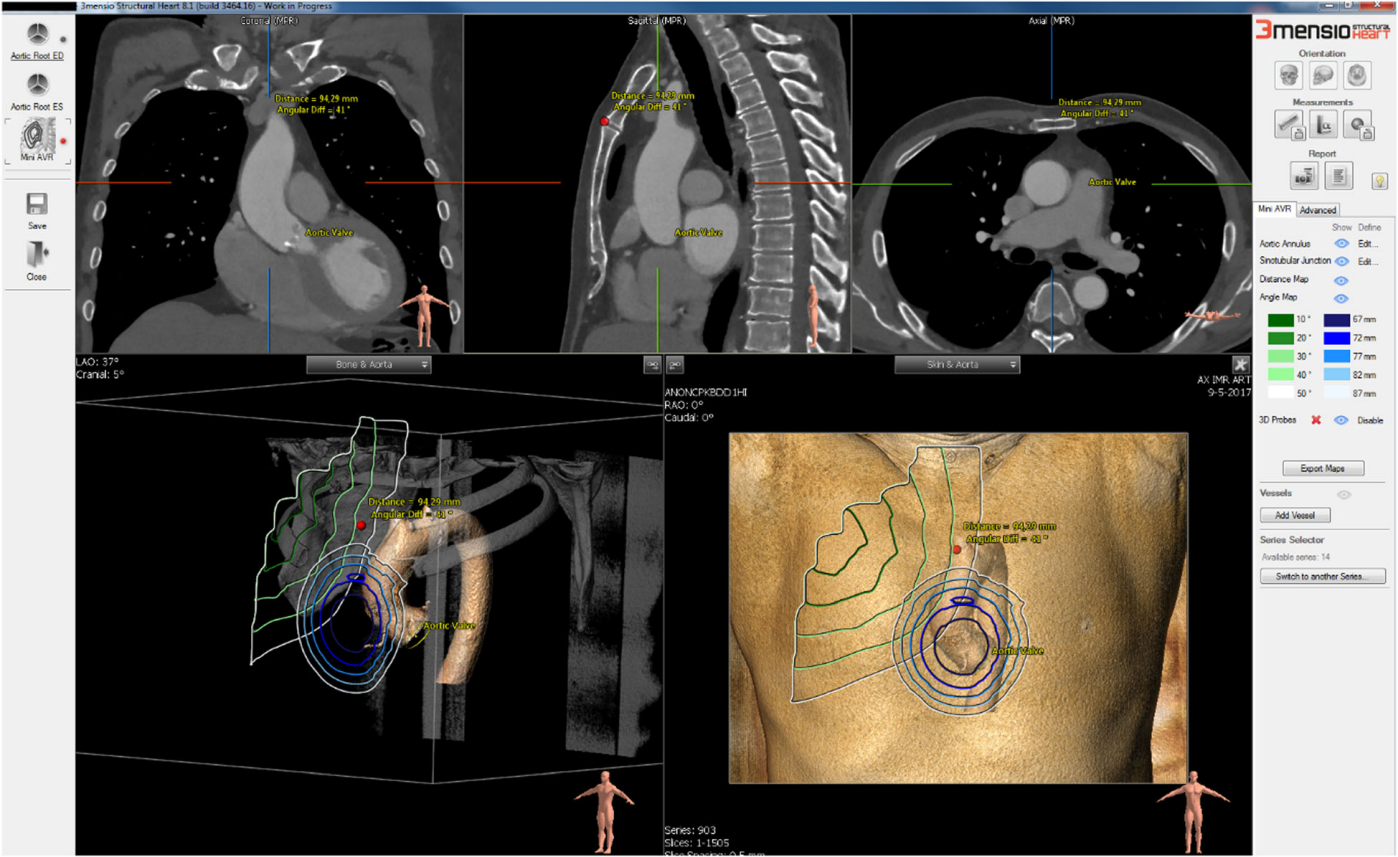
Screenshot of the mini-AVR planning tool. The graphical user interface (GUI) shows the isocontours of the access distance and access angle. The access angle contours are colorized ranging from dark green (small angle) to white (large angle). The access distance contours are colorized ranging from dark blue (short distance) to white (long distance). The quantitative measures are displayed in the legend on the right: the left column of bars for angles and right column for distance

We measured the access distance, which is the distance between the incision location on the chest and the aortic sinotubular junction. We also measured the access angle, which is the angle between the aortic root axis and incision-annulus axis (Fig. 1). The aortic root centerline axis is the centerline that runs from the annulus towards the sinotubular junction. The incision-annulus centerline is the centerline under which the surgeon views the exposed aortic valve. In addition, the tool also provides the access distance and access angle as isocontour maps rendered on the 3D rendered CT volumes (Fig. 2). To mark the location of the planned incision, a 3D probe can be placed at the center of the manubriosternal joint. The hinge points of the aortic valve leaflets are automatically detected allowing the determination of the annulus dimensions such as minimum, maximum diameters, area, and perimeter. Annulus measurements were only measured in CTA. We measured the calcium volume quantitatively in mm^3^ after thresholding the Hounsfield Unit intensities to separate the calcifications from the enhanced blood and aortic wall. The calcium volume is calculated after setting a volume of interest including aortic annulus and leaflet. Calcifications in the left ventricular outflow tract, coronary arteries and ascending aorta are excluded [23].

### Outcome parameters

Surgical complexity was assessed by the CPB time and AoX time as proxies for procedure complexity.

### Statistical analysis

Patients’ demographic and intra-operative data were expressed as mean (SD: standard deviation) for normally distributed values and as medians [interquartile range, IQR: 25th, 75th percentile] otherwise. Categorical data were presented as prevalence percentages.

Association of access distance, access angle, annular dimensions, and calcium volume with outcome parameters were tested with univariable linear regression.

Age and suture technique are known to also be associated with procedure complexity. Therefore, we adjusted for these confounders in multivariable analyses. The non-adjusted β_1_-coefficients and adjusted β_1_-coefficients with 95% Confidence Intervals (95%CI) of the linear regression models were presented.

To analyze whether there was a difference in the associations between sutured and sutureless valve replacement surgeries, an interaction term of the implantation technique and the CT-image based parameters that were significantly associated with the outcomes. The IBM SPSS Statistics, version 24 (IBM, Armonk, NY, USA) was used for statistical analysis.

## Results

One hundred patients that underwent mini-AVR and had a pre-operative CT scan were included in this study. The median age was 71 [IQR: 63, 75] years and 34 patients were female. Sixteen patients presented New York Health Association (NYHA) functional class III or IV symptoms. The median predicted operative risk by means of logistic EuroSCORE I was 4.3 [IQR: 2.9, 6.3]. Ten patients had mild left ventricular dysfunction, while the rest had normal left ventricular function. Eighty-three patients had severe aortic stenosis and 30 patients had a bicuspid aortic valve. Baseline characteristics, cardiovascular comorbidities and hemodynamic data are listed in Table 1.

**Table 1:** Baseline patient characteristics

Characteristics	Overall cohort (*n* = 100)
Age at surgery (years)	71 [63, 75]
Female gender	34 (34%)
Body mass index (kg/m^2^)	27 [25, 32]
NYHA Functional class ≥ III	16 (16%)
Cardiovascular risk factors	
Dyslipidemia	18 (18%)
Hypertension	54 (54%)
Diabetes mellitus	19 (19%)
Chronic obstructive pulmonary disease	12 (12%)
Peripheral vascular disease	5 (5.0%)
Previous cerebrovascular accident	9 (9.0%)
Previous transient ischemic attack	8 (8.0%)
Atrial fibrillation	22 (22%)
Severely renal impairment (CC ≤ 50)	3 (3.0%)
Previous history of percutaneous coronary intervention	10 (10%)
Logistic EuroSCORE I	4.3 [2.9, 6.3]
Pre-operative echocardiography	
Left ventricular function	
Mild dysfunction (LVEF 30–50%)	10 (10%)
Peak aortic transvalvular gradient (mmHg)	73 ± 22
Aortic Valve Area (cm^2^)	0.8 ± 0.2
Aortic valve pathology	
Severe aortic stenosis	84 (84%)
Aortic insufficiency grade ≥ III	4 (4.0%)
Aortic stenosis and insufficiency grade ≥ III	2 (2.0%)
Bicuspid aortic valve (BAV)	30 (30%)

Results are expressed as mean ± standard deviation, median [IQR: 25th, 75th percentile] or frequency (%). *CC* Creatine clearance, *EuroSCORE* European System for Cardiac Operative Risk Evaluation, *LVEF* Left ventricular ejection fraction, *NYHA* New York Heart Association

Fifty-five patients only underwent a CT and forty-five patients underwent both a CT and CTA. Ten scans were excluded for the access angle and access distance measurements: in these scan acquisitions the manubriosternal joint was not scanned, therefore it could not be used as a landmark for the incision location of our measurements. In total, 90 access distance and angle measurements were performed. For all patients the calcium volume was determined. Mean access angle was 42 (SD: 11) °, mean access distance was 86 (SD: 13) mm and the mean calcium volume was 689 (SD: 509) mm^3^. Forty-five annulus measurements were performed. The CT measurements are reported in Table 2.

**Table 2:** CT measured parameters

Variables	Mean ± standard deviation	Median [IQR: 25th, 75th percentile]
Access angle (degrees)	42 ± 11	40 [34, 49]
Distance from incision (mm)	86 ± 12	86 [77, 95]
Calcium volume (mm^3^)	6.9 · 10 ^2^ ± 5.1 · 10^2^	6.0 · 10^2^ [2.8, 9.2] · 10^2^
Annulus minimum diameter (mm)	23.8 ± 3.3	23.3 [21.4, 26.0]
Annulus maximum diameter (mm)	29.1 ± 4.1	27.8 [27.0, 30.5]
Annulus area (mm^2^)	5.5 · 10^2^ ± 1.8 · 10^2^	5.1 · 10^2^ [4.4, 6.1] · 10^2^
Annulus perimeter (mm)	8.5 · 10^1^ ± 1.2 · 10^1^	8.2 [7.0, 11] · 10^1^

CPB time and AoX time were 88 (SD: 24) and 63 (SD: 21) minutes, respectively. Ninety-nine patients had an isolated aortic valve replacement, with one mini-AVR procedure that was converted to a full sternotomy because the annulus could not be fully exposed to effectively perform the procedure. There was one patient that had a non-isolated aortic valve replacement, in which the patient required additional repair of the ascending aorta. Intra-operative data is summarized in Table 3.

**Table 3:** Intra operative data

Variables	Overall cohort (*n* = 100)
Isolated aortic valve replacement	99 (99%)
Conversion from mini-AVR to full sternotomy	1 (1.0%)
Non-isolated: Mini-AVR + Aortoplasty	1 (1.0%)
Sutureless valve prostheses	52 (52%)
Diameter of applied valve prosthesis	
21 mm	5 (5.0%)
23 mm	23 (23%)
25 mm	46 (46%)
27 mm	23 (23%)
29 mm	3 (3.0%)
Cardiopulmonary bypass time (min)	88 ± 24
Aortic cross-clamp time (min)	63 ± 21

Results are expressed as mean ± standard deviation or frequency (%)

In the univariable regression models, calcium volume, all annulus measures, sutureless valves, and age were significantly associated with CPB and AoX time (*p* < 0.05). Access angle and access distance were not associated with CPB and AoX time. Increasing levels of calcium volume and annulus measures showed an association with an increase in the CPB and AoX time, whereas advancing age and sutureless valves showed an association with a decrease in CPB and AoX time. After adjustment for confounding variables age and sutureless valves, only calcium volume was significantly associated with CPB (adjusted β-coefficient 0.002, 95%-CI (0.005, 0.019), *p*-value = 0.002) and AoX time (adjusted β-coefficient 0.010, 95%-CI (0.004, 0.016), *p*-value = 0.002) (Table 4).

**Table 4:** Univariable regression models of CT-based characteristics, age and suture technique with outcome measures. Linear regression models of CPB and AoX are presented with β-coefficients and confidence intervals (95%). In addition, for all CT-based characteristics the adjusted β-coefficients and confidence intervals (95%) are presented (corrected for age and suture technique). All values with a *p*-value < 0.1 are depicted in bold

	β-coefficient (95 CI%)	*P*-value	Adjusted β-coefficient (95 CI%)	*P*-value
Cardiopulmonary bypass time (min)				
Access angle (degrees)	−0.082 (− 0.56, 0.40)	0.74	− 0.071 (− 0.47, 0.32)	0.72
Distance from incision (mm)	0.15 (− 0.26, 0.57)	0.47	0.25 (− 0.096, 0.60)	0.15
Calcium volume (mm^3^)	**0.010 (0.001**, **0.019)**	**0.038**	**0.012 (0.005, 0.019)**	**0.002**
Annulus minimum diameter (mm)	**2.5 (0.5, 4.4)**	**0.013**	0.6 (−1.2, 2.3)	0.52
Annulus maximum diameter (mm)	**2.1 (0.5, 3.6)**	**0.010**	0.2 (−1.2, 1.6)	0.77
Annulus area (mm^2^)	**0.052 (0.013, 0.091)**	**0.011**	0.006 (−0.031, 0.042)	0.76
Annulus perimeter (mm)	**0.76 (0.21, 1.30)**	**0.008**	0.062 (−0.46, 0.58)	0.81
Sutureless valve (Yes)	**−28 (−36, −20)**	**< 0.001**		
Age (year)	**−0.96 (−1.44, − 0.48)**	**< 0.001**		
Aortic cross-clamp time (min)				
Access angle (degrees)	−0.090 (−0.50, 0.32)	0.66	−0.080 (− 0.40, 0.24)	0.62
Distance from incision (mm)	0.074 (− 0.28, 0.43)	0.68	0.17 (−0.11, 0.46)	0.23
Calcium volume (mm^3^)	**0.008 (0.000, 0.016)**	**0.051**	**0.010 (0.004, 0.016)**	**0.002**
Annulus minimum diameter (mm)	**2.5 (1.1, 3.9)**	**0.001**	1.0 (−0.2, 2.3)	0.10
Annulus maximum diameter (mm)	**2.2 (1.0, 3.3)**	**< 0.001**	0.8 (−0.2, 1.8)	0.13
Annulus area (mm^2^)	**0.055 (0.026, 0.083)**	**< 0.001**	0.021 (−0.006, 0.048)	0.12
Annulus perimeter (mm)	**0.79 (0.39, 1.18)**	**< 0.001**	0.289 (−0.094, 0.673)	0.14
Sutureless valve (Yes)	**−26 (−32, − 19)**	**< 0.001**		
Age (year)	**−0.93 (−1.32, 0.53)**	**< 0.001**		

There was no significant interaction found between suture technique and any of the annulus dimensions. Both suture technique and calcium were significant in the CPB and AoX models (*p* < 0.001), but their interaction coefficients were not (*p* = 0.24 for the CPB model and *p* = 0.15 for the AoX model).

## Discussion

This study shows that annulus dimensions and calcium volume are associated with CPB and AoX time, however, after adjusting for age and suture technique, the association between annulus dimensions and procedure times lost statistical significance. With increasing size in annulus dimensions and calcium volumes, CPB and AoX time increases. The use of sutureless valves and increasing age showed an association with a decrease in surgical time. In contrast to previous findings, in our population, access angle and distance were not associated with the procedure complexity and outcome.

In the study of Ellatar et al. [20], access angle was significantly associated with AoX time. We did not find such an association of access angle with neither AoX time nor CPB time. Our population is an extension of that same population, which could suggest that this previous finding was due to the limited experience in the procedure. In another study of Gilmanov et al. [2], access angle and distance were suggested to be associated with outcome parameters, which also contradicts with our findings. In their study, access angle and access distance were defined as the distance from ascending aorta to sternum and the angle between the sternum midline and position of the ascending aorta at the level of the main pulmonary artery. The discrepancy between these findings and the present study could be due to the differences in treatment selection. In our study, only patients that were treated through mini-sternotomy were included in this study, whereas the study of Gilmanov included mini-sternotomy and mini-thoracotomy procedures. Another reason why access angle and distance might not be associated with outcome parameters, is that the surgeons who performed the surgery have increased in skill over the years, and thus have become less dependent on the aortic anatomy. In Martella et al. [24], preoperative CT scans were used to plan the incision for right thoracotomies. Their results showed that if the incision location is perpendicular to the plane of the aortic valve, the surgeon has a better view on the exposed valve. It was shown that for this procedure, anterior and medially positioned aortas are more challenging because the angle towards the valve becomes more difficult, especially for right thoracotomies. Our study indicates that procedure complexity decreases with age. This may be explained by the fact that the elasticity of the aortic annulus and ascending aorta diminishes with advancing age. In contrast to our study, a previous research showed that increased annulus size was associated with a shorter procedural time, which was explained by the fact that the increased size in aortic annulus diameter and the elongation of the aorta gives the surgeon more working space [25]. This relation was not found in our study.

Our findings suggest that with each increment 100 mm^3^ of calcium surgical procedure time increases approximately by 1 min. So, a severely calcified aortic valve with an approximate volume of 500 mm^3^ would increase the CPB and AoX time by 5 min, which is a considerable amount of time since the average of our CPB and AoX time was 88 and 63 min respectively.

Surgical times are reduced by using sutureless valves, which has been confirmed by multiple studies [25–28]. This suggests that the choice of sutureless valves over sutured valves should always to be considered to decrease AoX time, in order to diminish the chance of post-operative morbidity and mortality [8, 10, 29]. In our study, the use of sutureless valves showed an association with a decrease of AoX time approximately by 26 min on average, which is a significant amount on an average of 63 min. Our study also suggests that there is no interaction between the annular dimensions and the choice of suture technique, indicating that the relation of annulus measures and calcium volume on surgical time is similar for both techniques.

Mini-AVR and CAVR are not the only choice of intervention for AS. The patient can also be treated through the less invasive transcatheter aortic valve implantation (TAVI). TAVI is favoured for high-risk and intermediate-risk patients who clinically are frail and old and have an increased risk for surgery. Mini-AVR and CAVR are favoured for low-risk and intermediate-risk patients, who might have endocarditis and might require additional interventions like revascularization of the coronaries. Surgery is considered when the aortic valve annulus is out of range for TAVI, the aortic root morphology is unfavourable for TAVI, and when the morphology of the valve (bicuspid valves, degree of calcification) is unfavourable for TAVI [30]. For the therapeutic choice between TAVI, mini-AVR and CAVR, a dedicated heart team assesses each patient based on previous cardiologic history and baseline characteristics, calculate the risk of surgery, evaluate the feasibility of TAVI or surgery, and local experience. TAVI is associated with increased pacemaker implantation, vascular complications and paravalvular leakage [31]. For mini-AVR and CAVR these complications are less common. However bleeding complications, acute kidney injury and new-onset atrial fibrillation happen more frequent when compared with TAVI [32]. When an institute is able to perform both surgical as percutaneous valve replacement, the heart team can evaluate technical suitability and risk-benefit ratio and decide the best course of action.

Our study suffers from a number of limitations: Only a single rater performed the CT measurements. The measures used to assess procedure complexity (CPB and AoX time) is only a derivative of the complexity and may under- or overestimate the surgical complexity. Although various steps of the measurements have been automated, which should reduce interobserver variation, the robustness of these measures and their association with the outcome parameters could be validated by more raters. The surgeries have been performed by multiple surgeons, which might have influenced the variability in surgical time. The image data was not uniform over the whole dataset. Non-contrast CT, CTA and multi-phase CTA were used based on what was available, because it is not a standard procedure to perform a CT before mini-AVR surgery. The number of bicuspid aortic valves was larger in the sutured group of patients, which might indicate bias to the selection of type of valve.

## Conclusion

We have shown that an increase in annulus size and calcium volume is associated with increased aortic cross-clamp and cardiopulmonary bypass time in patients with a mini-AVR. However, after adjusting for age and suture technique, the association between annulus dimensions and procedure times lost statistical significance.

Additionally, the study confirms that the implantation of sutureless valves and advancing age is associated with decreased aortic cross-clamp and cardiopulmonary bypass time. In contrast to previous studies, access angle was not associated with procedure complexity.

## Data Availability

The datasets used are available from the corresponding author on reasonable request.

## Abbreviations

AoX: Aortic cross-clamp
AS: Aortic stenosis
CAVR: Conventional aortic valve replacement
CC: Creatine clearance
CI: Confidence Interval
CPB: Cardiopulmonary bypass
CT: Computed tomography
CTA: Computed tomography angiography
EuroSCORE: European System for Cardiac Operative Risk Evaluation
GUI: Graphical user interface
ICU: Intensive care unit
IQR: Interquartile range
LVEF: Left ventricular ejection fraction
Mini-AVR: Minimal invasive aortic valve replacement
NYHA: New York Heart Association
SD: Standard deviation
TAVI: Transcatheter aortic valve implantation
